# Removal of a Large Steatomatous Tumor from the Neck

**Published:** 1831

**Authors:** John C. Brent

**Affiliations:** Shelbyville, Ky.


### Art. VI.—*Removal of a large Steatomatous Tumor from the Neck.* By John C. Brent, M. D., of Shelbyville, Ky.

*Cincinnati, Ohio, Jan.* 23, 1831.

Dear Sir—

Should you deem the following report, made on the day of operation, as meriting a place in your journal, it is at your disposal. The only important fact not embraced in the report, and which I now think proper to communicate, was the speedy recovery of the subject, aged 38 years, without the intervention of a single untoward symptom. Yours, most respectfully,

JOHN C. BRENT.

On the 18th of April last, having been called on by Mr. Samuel Graves, of Shelby Co., Kentucky, to examine a large tumor on the neck of his wife, of 12 years standing, and finding her constitution no further affected than might be expected from a tumor of such magnitude, I recommended its removal, and was, with the approbation of his lady, employed to perform the operation. The particulars, as detailed by Mrs. G., and indicated by the external appearances of the tumor, were the following: About 12 years ago, a small indurated body made its appearance near the middle of the omo hyoid muscle, but causing no pain, and enlarging slowly, it excited little alarm, until about 5 years ago, when its more rapid growth began to occasion much anxiety, and within the last 12 months, its increase has been estimated about one-third its previous size. External appearances exhibited the following anatomical limits: radiating from the point of commencement, its superior extremity was found nearly in contact with the mastoid process of the temporal bone, thence gradually sloping off, it covered about two inches of the superior face of the trapezins, passed over the origin of the deltoid, being pendulous over the whole extent of the right clavicle, crossed the sternum, and covered about 18 lines of the sternal extremity of the left clavicle, thence crossed the trachea obliquely, a few lines below the thyroid gland, and followed the course of the sterno cleido mastoideus, to its superior boundary. Neither the parotid, the thyroid, or any of the small lymphatic glands of the neck, seemed to be involved in the tumor, or was the great sheath containing the par vagum, the carotid artery, and jugular vein, much pressed in the erect position; but such was the weight of the tumor, that when horizontally placed, the patient experienced much inconvenience in respiration and deglutition, by its pressure on the trachea and œsophagus. Such was the thinness of the integuments, and the mobility of the tumor, (to all appearance,) as superficial as the platysma myoides, that I had no hesitation in advising its speedy removal, with the most encouraging hopes to the patient. On the 8th of May, after all necessary preparations, the patient, with great composure, submitted to this formidable operation, in the presence of several medical students from Shelbyville, and many friends of the family who attended on the occasion, and bore it with unusual fortitude and steadiness. But six important blood vessels were divided, and these through the coolness and intrepidity of Dr. Wilson, of Shelbyville, whose services are most thankfully acknowledged, I was enabled so promptly to secure by ligature, as entirely to obviate exposure to syncope.

*Dimensions.*—Large diameter 6 1-2 inches.
Short dameter 5 do.
Circumference 21 do.
Weight about 3 pounds.

